# Explicitly modeling genetic ancestry to improve polygenic prediction accuracy for height in a large, admixed cohort of US Latinos: Findings from HCHS/SOL

**DOI:** 10.1016/j.xhgg.2026.100597

**Published:** 2026-03-27

**Authors:** Xin Wang, Tamar Sofer, Oleksandr Frei, Robert Kaplan, Krista M. Perreira, Nora Franceschini, Humberto Parada, Laura Zhou, Ole A. Andreassen, Hector Gonzalez, Anders M. Dale, Iris J. Broce

**Affiliations:** 1 Department of Neurosciences, University of California, San Diego, San Diego, CA 92093, USA; 2 Cardiovascular Institute, Beth Israel Deaconess Medical Center, Harvard Medical School, Boston, MA 02215, USA; 3 Department of Biostatistics, Harvard T.H. Chan School of Public Health, Boston, MA 02215, USA; 4 Department of Medicine, Brigham and Women’s Hospital, Boston, MA 02215, USA; 5 Institute of Clinical Medicine, University of Oslo, 0316 Oslo, Norway; 6 NORMENT, K.G. Jebsen Centre for Psychosis Research, Institute of Clinical Medicine, University of Oslo, 0316 Oslo, Norway; 7 Department of Epidemiology and Population Health, Albert Einstein College of Medicine, Bronx, NY 10461, USA; 8 Public Health Sciences Division, Fred Hutchinson Cancer Center, Seattle, WA 98109, USA; 9 Department of Public Policy, University of North Carolina at Chapel Hill, Chapel Hill, NC 27599, USA; 10 Department of Epidemiology, University of North Carolina, Chapel Hill, NC 27599, USA; 11 Division of Epidemiology and Biostatistics, School of Public Health, San Diego State University, San Diego, CA 92182, USA; 12 Department of Biostatistics, University of North Carolina, Chapel Hill, NC 27599, USA; 13 Weill Institute for Neurosciences, Department of Neurology, University of California, San Francisco, San Francisco, CA 94143, USA

**Keywords:** polygenic scores, genetic ancestry, admixed population, UK Biobank, Hispanic Community Health Study/Study of Latinos, height prediction, principal components

## Abstract

Polygenic scores (PGSs) offer moderate to high prediction accuracy for complex traits, but most are developed in European ancestry cohorts, reducing their performance in populations of other ancestries. This study aimed to improve standing height prediction, a heritable and ancestry-influenced trait, in an admixed Latino cohort, the Hispanic Community Health Study/Study of Latinos (HCHS/SOL), by modeling ancestry using principal components (PCs) alongside PGSs. SNPs were selected from a large European ancestry genome-wide association study (GWAS) using various *p* value thresholds, and weights were trained using traditional and penalized regression in the UK Biobank (UKB). PGSs with PCs were trained separately in the HCHS/SOL and UKB. Compared to PGSs alone, modeling PGSs with PCs moderately improved height prediction in the HCHS/SOL (squared correlation [R^2^] increase of ∼0.05), while mild improvements were observed in the UKB (R^2^ increase of ∼0.01). These results underscore the importance of incorporating genetic ancestry into predictive models for admixed populations, particularly when the trait exhibits ancestry-specific associations.

## Teaser

Polygenic scores (PGSs) are predominantly derived from European cohorts and often fail to generalize to admixed populations. Using standing height, we show that incorporating genetic ancestry alongside PGSs improves prediction accuracy in an admixed Latino cohort, underscoring the need for ancestry-informed modeling to enhance performance in diverse populations.

## Introduction

Genome-wide association studies (GWASs) have identified common genetic variants that influence susceptibility to complex traits and diseases. Polygenic risk models aggregate these small-to-moderate contributions to create polygenic scores (PGSs) that provide improved predictive accuracy for quantitative traits and disease risk relative to any individual genetic marker.^1^^,^^2^ Because the majority of GWASs and subsequent polygenic prediction analyses have been conducted on cohorts predominantly of European ancestry, the generalizability of PGSs to non-European and admixed populations is reduced.^3^^,^^4^^,^^5^^,^^6^ Studies within the Hispanic Community Health Study/Study of Latinos (HCHS/SOL) and others have shown that PGS performance depends on how closely the ancestry of the study population is to the group used to develop the score.^3^^,^^7^^,^^8^ As sample sizes of non-European cohorts increase with more diverse and generalizable populations, the potential for discovery and prediction will improve.^9^ However, practical interim approaches are needed to improve accurate predictions across diverse ancestries, thereby mitigating disparities in research outcomes.

The predictive accuracy of PGS for a trait depends in part on its SNP heritability. Standing height serves as a prime example of a highly heritable, polygenic trait, with heritability based on twin studies of approximately 70%–90%^10^^,^^11^ and SNP heritability of about 40%–50%.^8^^,^^12^ High SNP heritability indicates that a significant portion of the trait’s variation is due to the additive effects of SNPs. The SNP heritability provides a quantitative estimate of the asymptotic prediction performance using linear prediction as the sample size approaches to infinity.^5^ The difference between the higher twin heritability and the SNP heritability is commonly referred to as “missing heritability” and has been hypothesized to be due to rare genetic variants, structural variants, epistatic effects, and/or gene-environment interactions.^13^^,^^14^ Thus, while SNP heritability sets a limit on the accuracy of PGS predictions and, as sample sizes grow, these predictions may approach this theoretical maximum performance, factors such as rare variants and non-additive effects can hinder PGS from fully realizing its potential.

An important limitation of current polygenic prediction methods is their poor generalization performance across ancestries.^3^ PGSs often exhibit reduced accuracy in populations with ancestries that differ from those used in the training GWAS due to differences in allele frequencies, linkage disequilibrium (LD) patterns, and/or causal effect sizes.^3^^,^^4^ Factors influencing differences in allele frequencies and LD patterns include genetic factors influenced by natural selection, historical events such as colonization and forced migration, voluntary migration, and mating strategies.^15^ Genetic principal components (PCs) provide a straightforward approach to capturing genetic structure patterns across ancestries. Adjusting for PCs in GWASs is important to prevent false-positive associations lacking biological relevance and to ensure that ancestry information does not affect the weight of individual SNP effects. However, some evidence suggested that when applying PGS to a validation cohort with ancestry different from the training cohort or GWAS, explicitly modeling ancestry information within that validation cohort can be beneficial for improving prediction accuracy.^16^^,^^17^ One study applied this approach and demonstrated improved PGS prediction in European subgroups, including those with Scandinavian, Southern European, and Ashkenazi Jewish ancestry, for three pigmentation-related traits: natural hair color, childhood and adolescent tanning ability, and basal cell carcinoma.^16^ This strategy is thought to enhance prediction accuracy by accounting for genetic structure and compensating for variations in allele frequencies and LD that might not be adequately represented in the training cohort.^16^ However, the extent to which explicitly modeling ancestry improves polygenic prediction accuracy in non-European, admixed individuals—such as Latinos with varying proportions of Amerindian, European, and African ancestry—remains unclear.

To better understand this, we explored how incorporating PGSs with PCs affects the accuracy of polygenic predictions of standing height compared to using PGSs alone. Our analysis focused on a European descent training cohort from the UK Biobank (UKB) and a highly admixed Latino validation cohort, specifically the HCHS/SOL, which includes individuals with diverse ancestry proportions.^18^ We used the latest GWAS summary statistics for height^8^ to select SNPs. Standing height was chosen as the trait of interest due to its high heritability and known association with ancestry. In the European cohorts from which these GWAS summary statistics were derived (including and excluding the UKB), SNP-based heritability has been estimated at approximately 0.45–0.50, supporting the suitability of height as a benchmark phenotype.^8^^,^^9^ To validate our results and minimize overfitting, we employed cross-validation schemes and used squared correlation [R^2^] to assess predictor efficacy, particularly the impact of explicitly modeling ancestry. To construct polygenic predictors, we used penalized regression methods, which estimate SNP effects simultaneously while applying regularization to mitigate multicollinearity and reduce overfitting. Specifically, Lasso regression shrinks coefficients based on their absolute values, often reducing some coefficients to exactly zero. This property makes it effective for SNP selection by eliminating less relevant predictors. Ridge regression shrinks coefficients based on their squared values, controlling for large coefficients. These methods have demonstrated superior performance to conventional PGS approaches across diverse populations.^19^

## Subjects and methods

### UKB

The UKB is a large-scale prospective epidemiological study of over 500,000 individuals aged 40–69 years from the United Kingdom, which was established to investigate the genetic and non-genetic determinants of middle- and old-age diseases (https://www.ukbiobank.ac.uk/). All participants provided written informed consent, and ethical approval for the UK Biobank was obtained from the North West Multicenter Research Ethics Committee.

The UKB released genetic data for 487,409 individuals in 2018 (v.3). These samples were genotyped using either Affymetrix UK BiLEVE Axiom or Affymetrix UKB Axiom arrays (Santa Clara, CA, USA), which include over 800,000 genetic variants. The UKB researchers implemented extensive quality control (QC) procedures on genotype data.^20^ Imputation was performed centrally using combined data from the 1000 Genomes Project and the UK10K panel,^21^ with SHAPEIT3 used for phasing and IMPUTE2 used for imputation.^22^ Genotype QC was performed using PLINK v.2.00a3.6LM (August 14, 2022). For each chromosome, we applied the following filters: genotype call rate < 10%, Hardy-Weinberg equilibrium *p* < 1 × 10^−10^, and minor-allele frequency < 0.001. Individuals flagged for withdrawal by the UKB were removed prior to analysis. These QC criteria resulted in the inclusion of 11,076,367 SNPs. Forty PCs of genetic ancestry were supplied by the UKB (field 22009).

Standing height (cm) was supplied by the UKB (field 50). In this study, we included 485,638 individuals with different ethnic backgrounds (referred to as “UKB ALL”) with height measurements and genotype data after excluding height outliers (see below). 407,843 participants of European ancestry (referred to as “UKB EUR”) who self-identified as “White British” (field 21000) and exhibited similar genetic ancestry based on principal-component analysis (PCA) of their genotypes were included in the analyses.

### The HCHS/SOL

The HCHS/SOL^18^^,^^23^^,^^24^ is a population-based longitudinal cohort that follows Hispanic/Latino participants from four metropolitan areas, Bronx, NY; Miami, FL; Chicago, IL; and San Diego, CA, with 16,415 participants aged 18–74 years examined at the baseline visit. Participants were recruited using a two-stage area probability sampling design across the four metropolitan areas in which households were randomly sampled within selected census block groups, and all Hispanic/Latino adults aged 18–74 years were invited to participate. Additional details on the HCHS/SOL study design are provided in Sorlie et al.^18^ and Lavange et al.^23^ The HCHS/SOL protocol was approved by each local institutional review board, and all participants provided informed consent. A total of 12,803 study participants consented to genetic studies.^6^^,^^23^ Participants self-identified with six Hispanic/Latino background groups: Central American, South American, Mexican (mainland groups; have high Amerindian genetic ancestry and low African ancestry), Cuban (high proportion of European ancestry, low proportions of African and Amerindian ancestry), Dominican, and Puerto Rican (Caribbean group; have low Amerindian ancestry and high African ancestry proportions).^25^^,^^26^ In this study, we included 11,856 individuals who participated in the HCHS/SOL study and were genotyped. All individuals provided written informed consent at their recruitment site.

### Genotyping

HCHS/SOL genetic data were assessed from blood during the baseline exam. Samples have been genotyped using three versions of the biobank SNP array offered by Illumina, which is designed to capture the diversity of genetic backgrounds across the globe. The first batch of data was generated on the Multi-Ethnic Genotyping Array (MEGA) array, the first release of this SNP array. The second, third, and fourth batches were generated on the Expanded MEGA (MEGA Ex) array. All remaining data were generated on the Multi-Ethnic Global (MEG) BeadChip.^27^^,^^28^ Individuals who consented to genetic studies were genotyped using the Illumina MEGA array, and a total of 11,928 samples and 985,405 genotyped variants passed QC. For additional details about the DNA sampling, see Conomos et al.^26^ and Sofer et al.^27^^,^^28^

Genotype data were imputed to the Trans-Omics in Precision Medicine (TOPMed) freeze 5b reference panel as described previously.^29^ QC procedures in the HCHS/SOL, including methods used to construct the kinship matrix reflecting genetic relatedness between study participants (some of whom were sampled from the same household), have been described previously.^26^ PCAs were performed using FlashPCA2.^30^^,^^31^ Global ancestry proportions, measuring the proportion of the genome inherited from European, African, and Amerindian ancestors, and genetic PCs were computed as previously reported.^26^ Continental ancestry proportions were estimated using a model-based approach with ADMIXTURE software,^32^ assuming the presence of three ancestral populations (African, European, and Amerindian for k = 3).^26^ We classified the participants into three ancestry groups based on the maximum value among the continental ancestry proportions.

In this study, we included 11,856 genotyped participants from the HCHS/SOL. Individuals with extreme height outlier values were excluded, as detailed below. All individuals provided written informed consent at their recruitment site.

### Summary statistics from published GWASs

Summary statistics were derived from the largest and most recent GWAS, which included data from 5.4 million individuals and identified over 12,000 genetic variants associated with variation in human height.^8^ In this study, we used GWAS summary statistics of Europeans, including or excluding the UKB (https://portals.broadinstitute.org/collaboration/giant/index.php/GIANT_consortium_data_files).

### Statistical analyses

#### PGS development

Standing height was first residualized over age, sex, and 40 PCs. Standardized residuals were then computed, and participants with |z| ≥ 4 were excluded as extreme outliers. We selected this threshold based on previous studies using similar criteria for phenotypic exclusion in the UKB cohort.^33^ In our sample, this resulted in the exclusion of *n* = 128 in UKB EUR, *n* = 156 in UKB ALL, and *n* = 8 in the HCHS/SOL. Following exclusion, residualized height was re-standardized (mean = 0, SD = 1) for subsequent analyses.

We divided the European subset of the UKB cohort into 90% for training (*n* = 367,059) and 10% for validation (*n* = 40,784). We validated the PGS in the HCHS/SOL as well (*n* = 11,856) (Figure S1). The training cohort was used to estimate SNP effects, and the resulting weights (coefficients) were applied to the validation cohorts. Penalized regression models were fit using the “glmnet” function in MATLAB, where the dependent variable was the standardized phenotype and the independent variable was an *N* × M matrix of standardized genotypes (*N*, number of participants; M, number of SNPs).

Our PGS calculation is based on a regularized regression framework, which differs from the classical clumping-and-thresholding (C+T) approach. For example, the Lasso regression (α = 1) directly estimates SNP effects while penalizing correlated predictors by shrinking redundant SNP effects toward zero, while ridge regression (α = 0) shrinks coefficients toward zero without excluding SNPs. Both methods provide regularization that helps account for LD among predictors, eliminating the need for explicit LD pruning or a separate C+T procedure. This strategy is consistent with previous work by our group.^34^

We selected SNPs for model training from publicly available GWAS summary statistics for standing height in European ancestry cohorts, using versions both excluding and including the UKB.^8^ Specifically, when model training and validation were both conducted within the UKB (UKB EUR), we used GWAS summary statistics from European ancestry cohorts that excluded the UKB to avoid overfitting. When training was performed in the UKB and prediction in the HCHS/SOL, we used European ancestry GWASs that included the UKB to increase the training sample size. To ensure a systematic comparison, we repeated the HCHS/SOL prediction using GWASs that excluded UKB.

Given the sensitivity of penalized models to missing data, we restricted analyses to SNPs present across GWAS summary statistics, the UKB, and the HCHS/SOL cohorts. Ambiguous SNPs were excluded from the analyses. We assessed models using a range of *p* values: 1 × 10^−16^, 1 × 10^−12^, 1 × 10^−8^, 1 × 10^−4^, 1 × 10^−3^, 1 × 10^−2^, and 1 × 10^−1^. Corresponding SNP counts are provided in Table S1.

When fitting the penalized regression model, we set the alpha parameter of the glmnet package to 1 for Lasso regression and 0 for ridge regression. The model used 10 lambda values, resulting in 10 sets of SNP weights. The lambda values were selected based on the best prediction accuracy of the corresponding PGS (with the highest R^2^). We constructed 10 PGSs (one per lambda) for individuals in the UKB validation cohort and the HCHS/SOL cohort by multiplying the number of risk alleles for each SNP by its corresponding weight and summing these weighted scores across all SNPs (Equation 1). The 10 lambda values are provided in Table S2.

(Equation 1) $$\text{PGS}=\sum_{j=1}^{M}\beta j*g_{j}\text{,}$$

where M is the number of SNPs, β_j_ is the weight of j-th SNP effect allele, and *g*_*j*_ is the effect allele count of the j-th SNP.

### PGS prediction with PCs

The same residualized and standardized height phenotype was used for both PGS-only and PGS-with-PCs prediction analyses. Standing height was first adjusted for age and sex, then standardized, and participants with |z| ≥ 4 were excluded as extreme outliers.

To estimate the PGS-only prediction, the R^2^ between 10 PGSs and the standardized phenotype was computed, prediction accuracy was quantified as the highest R^2^, and the best-performing PGS (highest R^2^) was used in analyses. To estimate the predictive contribution of ancestry components in addition to genetic effects, we implemented a two-stage validation approach (Figure S1C). Specifically, for both the UKB validation cohort (*n* = 40,784) and the HCHS/SOL (*n* = 11,856), we performed a secondary random split of 80% (training) and 20% (validation). Linear regression models were fitted separately in the 80% subset within each cohort (UKB EUR: *n* = 32,627; HCHS/SOL: *n* = 9,485), with standardized height as the outcome and both PGSs and PCs as predictors. This allowed PC effects to be estimated independently of the subset used to evaluate prediction performance.

The model coefficients for PGSs and PCs from the linear model were then applied to the 20% validation cohorts (UKB EUR: *n* = 8,157; HCHS/SOL: *n* = 2,371) to generate predicted height (see Equation 2, using the top 5 PCs as an example). Prediction accuracy was quantified as the R^2^ between observed standardized and predicted heights. To ensure robustness given the reduced sample size in each split, this procedure was repeated across 1,000 random 80/20 partitions, and the final R^2^ value was calculated as the mean across all iterations.

(Equation 2) Y=β1∗PGS+β2∗PC1+β3∗PC2+β4∗PC3+β5∗PC4+β6∗PC5

β1 is the coefficient of PGS and β2–β6 are the coefficients of the top 5 PCs, respectively. Y is the predicted standardized sex- and age-adjusted height.

Since the HCHS/SOL includes multiple family members, there is a risk that relatives appearing in both the training and validation cohorts could artificially inflate the prediction accuracy. To mitigate this risk, we ensured that individuals with third-degree or closer familial relationships, as identified by kinship values greater than 0.0442, were included only in the testing group. We also applied the PGS trained on UKB EUR to different predominant ancestry subsets of the HCHS/SOL (Figure S2).

## Results

### Participants

**Figure 1:**
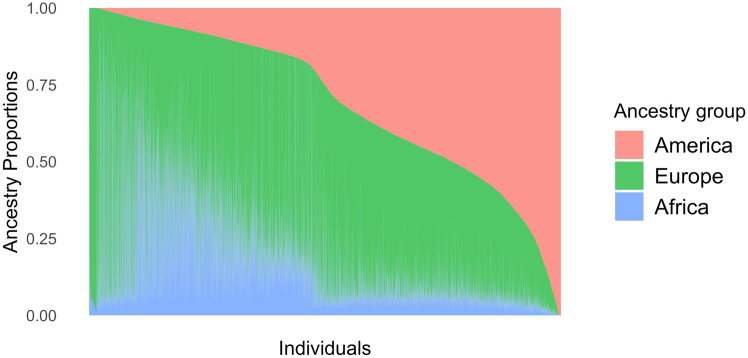
Varying proportions of genetic ancestry from three continental regions for HCHS/SOL participants

We used two main cohorts for our analysis: 407,843 participants of UKB EUR and 11,856 participants from the HCHS/SOL,^18^ all with available genotype and phenotype data. Demographics are shown in Table 1. Figure 1 illustrates the varying proportions of genetic ancestry from three continental regions for the HCHS/SOL: European, Amerindian, and African (see subjects and methods). Continental ancestry proportions were estimated using a model-based approach with ADMIXTURE software,^32^ assuming the presence of these three ancestral populations (African, European, and Amerindian for k = 3).^26^ Participants were classified into predominant ancestry groups based on the maximum value among the continental ancestry proportions.

**Table 1:** Demographics of participants

	UKB EUR	UKB ALL	HCHS/SOL
*N*	407,843	485,638	11,856
Age (year)	57.41 (8.0)	57.04 (8.09)	46.08 (13.83)
Sex, women, *N* (%)	220,494 (54.1%)	263,444 (54.3%)	6,950 (58.6%)
Standing height (cm)	168.67 (9.23)	168.43 (9.26)	162.12 (9.29)

In supplementary analyses, we examined the UKB ALL, which includes an additional 77,795 participants from diverse ethnic backgrounds, including those of non-European origins. This was done to assess whether this inclusion improved the generalizability of our findings in the HCHS/SOL.

### Mild improvement in prediction accuracy by modeling PCs with PGSs compared to PGSs alone when all training, validation, and GWAS cohorts are of European ancestry

We selected height-associated SNPs from the most recent and largest publicly available GWAS summary statistics for standing height derived from a cohort of European ancestry (excluding UKB individuals)^8^ (Figure S1). The number of SNPs used to construct PGSs under different *p* value thresholds is summarized in Table S1 (see subjects and methods). We split the UKB EUR cohort, using 90% of samples (*n* = 367,059) to develop the PGS with Lasso and ridge penalized regression models and allocating 10% of samples (*n* = 40,784) for validation. When modeling linear combinations of PGSs with PCs, we further divided the UKB EUR validation cohort, allocating 80% (*n* = 32,627) for training and 20% for validation (*n* = 8,157).

**Figure 2:**
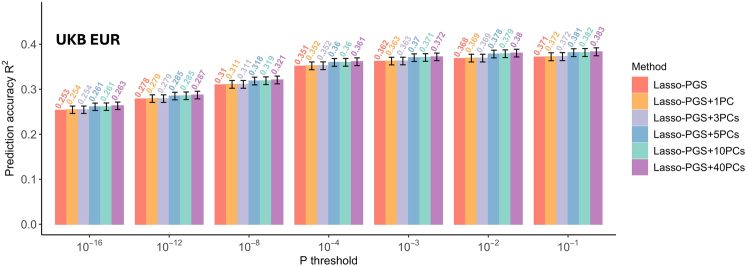
Comparison of predictive performance for height in the UKB EUR validation cohort between using PGSs alone and a combination of PGSs and PCs Error bars indicate SD. UKB EUR, UK Biobank European; PGSs, polygenic risk scores; PCs, principal components.

Using Lasso-derived PGSs, when the PGS for height was included in the prediction model without PCs (PGS only), prediction accuracy (R^2^) in the UKB EUR validation cohort improved incrementally as more SNPs were included at higher *p* value thresholds (Figure 2). Specifically, the PGS R^2^ was 0.253 when including SNPs that met a 10^−16^ *p* value threshold and improved to 0.371 when including SNPs that met a 10^−1^ *p* value threshold (Figure 2). When PCs were added to the prediction model, R^2^ increased slightly with each additional PC, showing a mild improvement of 0.01 in R^2^ across all *p* value thresholds (Figure 2). The highest R^2^ reached 0.383 at 10^−1^ *p* value thresholds while including 40 PCs. We also trained PGSs using ridge regression and observed similar trends as with Lasso regression (Figure S3).

### Moderate improvement in prediction accuracy by modeling PCs with PGSs compared to PGSs alone when the validation cohort consists of admixed Latino individuals and when training and GWAS cohorts are of European ancestry

For these analyses, we selected height-associated SNPs from the full EUR GWAS summary statistics for standing height (i.e., including the UKB), as the HCHS/SOL was not part of the original GWAS discovery sample (see subjects and methods; Figure S1). The number of SNPs used to construct PGSs under different *p* value thresholds is summarized in Table S1. We developed the PGS using the UKB EUR via Lasso and Ridge penalized regression models (as above). We then applied the SNP weights derived from this model to the entire HCHS/SOL cohort. To model the PGSs with PCs, we divided the HCHS/SOL cohort into a training set (80%, *n* = 9,485) and a validation set (20%, *n* = 2,371).

**Figure 3:**
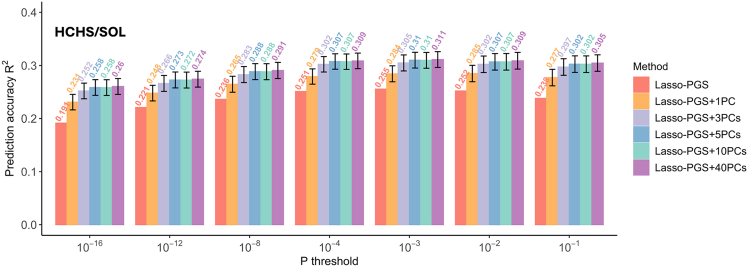
Comparison of predictive performance for height in the HCHS/SOL between using PGSs alone and a combination of PGSs and PCs Error bars indicate SD. PGS, polygenic risk score; PCs, principal components.

Using Lasso-based PGSs, when only a PGS for height was included in the prediction model, the prediction accuracy (R^2^) varied across different *p* value thresholds. The lowest R^2^ (0.191) was observed when the fewest SNPs were incorporated at a *p* value threshold of 10^−16^, while the highest R^2^ (0.255) was observed at a *p* value threshold of 10^−3^ (Figure 3). Including PCs in the prediction model boosted the accuracy of height prediction, improving the R^2^ by roughly 0.05–0.07 across all *p* value thresholds. The highest prediction accuracy achieved was 0.311, observed at thresholds of 10^−3^ with the inclusion of 40 PCs. The greatest enhancement in predictive performance consistently occurred with the inclusion of the first 3 PCs across the different *p* value thresholds (Figure 3). Beyond the first 3 PCs, improvements were minimal, consistent with previous findings suggesting that the first 2–3 PCs capture the majority of underlying variance.^16^

We performed two sensitivity analyses. First, to assess whether improvements from modeling genetic ancestry could be partly driven by recruitment site, which in the HCHS/SOL is known to correlate with self-identified background and ancestry composition,^26^ we compared PGS+PC linear models with and without including the center as a covariate. Across 1,000 iterations, the mean prediction accuracy was R^2^ = 0.3014 when the recruitment center was included vs. R^2^ = 0.2824 without the center (ΔR^2^ = 0.019). Thus, the recruitment site explained only ∼1.9% of the variance beyond that captured by PGSs and PCs, indicating that the improvement in prediction accuracy is largely attributable to genetic ancestry effects rather than non-genetic site-related factors.

Second, for consistency, we repeated the HCHS/SOL prediction analyses using GWAS summary statistics excluding the UKB, applying the same SNP selection procedure as in the UKB EUR analyses. Prediction accuracy was slightly lower than using the saturated GWAS including the UKB, but the improvement from incorporating PCs was consistent (Figures 2 and S4). These results indicate that modeling genetic ancestry enhances prediction in admixed populations irrespective of GWAS reference size. Accordingly, GWAS summary statistics that included the UKB were used for all subsequent HCHS/SOL analyses.

Finally, to verify that our findings were not specific to the choice of penalization method, we trained PGSs using ridge regression and obtained similar results showing that incorporating PCs significantly increased the prediction accuracy in the HCHS/SOL (Figure S3).

### Across all three predominant ancestry groups in HCHS/SOL, the most significant improvement in predictive performance was achieved by including the first 3 PCs

We stratified the HCHS/SOL participants into three predominant ancestry groups based on their highest estimated ancestry proportion, European (*n* = 6,367), Amerindian (*n* = 2,764), and African (*n* = 733), and investigated the prediction accuracy within each group separately (Figures S2–S5). As expected, given the European-only GWAS reference, the PGS-only model achieved its highest performance in the European-predominant subgroup, with a maximum R^2^ = 0.253 at a *p* value threshold of 10^−2^. Including PCs improved prediction accuracy by approximately 0.01 (maximum R^2^ ≈ 0.264). In the Amerindian-predominant subgroup, the PGS-only model showed lower baseline accuracy (maximum R^2^ = 0.230 at *p* = 10^−3^), but incorporating PCs resulted in a larger improvement of approximately 0.08, with the maximum R^2^ increasing to 0.313. The higher R^2^ observed in the Amerindian-predominant subgroup when PCs were included likely reflects the additional variance explained by genetic ancestry, as PCs capture ancestry-specific structure that contributes to height variation in this admixed cohort. In the African-predominant subgroup, prediction accuracy was more variable, most likely due to the smaller sample size resulting in larger error bars. The PGS-only model reached a maximum R^2^ of 0.128 at *p* = 10^−4^. Including PCs improved prediction by approximately 0.03, with the maximum R^2^ increasing to 0.162. Across all three ancestry groups, the most significant improvement in predictive performance was consistently achieved by including the first 3 PCs (Figure S5).

### Increasing the training sample size by including individuals of varied ancestry enhanced out-of-sample prediction accuracy in the HCHS/SOL only when ancestry is explicitly modeled

Following the same methods as above, we re-trained our PGS and PGS with PCs models using the UKB ALL as a training cohort and applied these weights to both the UKB ALL validation and HCHS/SOL cohorts (Figures S6 and S7).

When models were applied to UKB ALL, the highest prediction accuracy was observed in the PGS+PC setting with 40 PCs (maximum R^2^ = 0.396), similar to the models trained in UKB EUR (maximum R^2^ = 0.383). In contrast, when models were applied to the HCHS/SOL, PGS-only accuracy was lower when trained in UKB ALL compared with UKB EUR (maximum R^2^ = 0.145 vs. 0.255). Notably, because height was not residualized on PCs for these analyses, this likely reflects residual population stratification in the UKB ALL training set, reducing generalizability to the admixed HCHS/SOL cohort. However, once genetic ancestry was explicitly modeled using PCs, prediction accuracy became comparable for models trained in UKB ALL relative to UKB EUR (maximum R^2^ = 0.315 vs. 0.311). These results suggest that increasing ancestry diversity in the training cohort does not inherently improve generalization to admixed populations unless genetic ancestry is explicitly modeled.

We also applied PGSs trained in UKB ALL to the different predominant ancestry subgroups in the HCHS/SOL (Figures S8). Across subgroups, prediction accuracy was similar to models trained in UKB EUR, with the largest improvements consistently achieved only when PCs were explicitly included. Notably, despite the greater ancestral diversity in the UKB ALL training cohort, this did not result in improved PGS-only performance in the Amerindian-predominant subgroup. This may reflect the overall representation of Amerindian genetic structure in UKB ALL. Therefore, the gains observed in the HCHS/SOL appear to arise primarily from explicit modeling of genetic ancestry via PCs rather than from increased ancestry diversity in the training data alone.

## Discussion

This study evaluated how modeling genetic ancestry information, through PCs, alongside PGSs impacts the prediction accuracy of standing height, with a focus on improving the generalizability of PGSs to non-European and admixed populations. We used the most recent and largest GWAS summary statistics for standing height to select SNPs and trained the PGSs within the UKB using Lasso and ridge penalized regression methods. We applied these PGSs to both the UKB and the HCHS/SOL, an admixed cohort of Latino individuals. We assessed prediction accuracy using the R^2^ metric. Collectively, our results demonstrated that explicitly modeling ancestry information with PGSs improves height prediction performance in the HCHS/SOL compared to using PGSs alone. Importantly, our results suggest that increasing ancestry diversity in the training cohort alone does not guarantee improved prediction accuracy in admixed populations. Rather, prediction was highest when the ancestry composition of the training cohort more closely matched that of the target population and when genetic ancestry was explicitly modeled to capture relevant structure. Models trained in European-only cohorts performed best when applied to individuals with predominantly European ancestry, highlighting that current predictive models are still the most accurate when the training and target populations are genetically similar. Therefore, continued data collection in diverse populations is critical to improving model generalizability and reducing disparities in prediction accuracy.

Previous studies have shown that explicitly modeling ancestry, even within European subgroups (Scandinavian, Southern European, and Ashkenazi Jewish ancestry), can enhance the predictive performance by R^2^ of 0.02–0.03 for ancestry-associated traits, such as hair color, tanning ability, and basal cell carcinoma.^16^ In our European UKB cohort, we observed slight prediction improvements (R^2^ = ∼0.01, depending on *p* value thresholds) when modeling ancestry with PGSs. The mild gain is likely due to the cohort’s pre-selection of White British individuals with PCs confirming European ancestry, minimizing ancestry-related genetic variation. However, when the training and validation cohorts were from different ancestral backgrounds—such as applying PGSs derived from a UKB EUR cohort to the HCHS/SOL—modeling ancestry information within the HCHS/SOL alongside PGSs moderately enhanced prediction accuracy (R^2^ = 0.05–0.07). Collectively, these findings suggest that if the goal is to improve prediction, adding ancestry information can be beneficial, particularly when the training and validation cohorts differ in ancestry and the trait of interest is ancestry dependent.

The generalizability of PGSs across ancestries remains an active area of investigation, and several recent studies have proposed strategies to address this challenge. For example, Bitarello and Mathieson^35^ showed that PGS accuracy for height declines with decreasing European ancestry and that incorporating ancestry-specific effect estimates—by recalibrating SNP weights within ancestry components or accounting for local ancestry—can partially restore prediction performance in admixed populations. Lehmann et al.^36^ demonstrated that using multi-ancestry or ancestry-matched training sets can improve prediction relative to large European-only GWASs, emphasizing the role of sample composition in model generalizability. Our results extend this line of work by showing that incorporating genetic ancestry directly into prediction models enhances PGS performance in admixed populations.

We found that the highest prediction accuracy for height in the HCHS/SOL did not match the peak accuracy achieved in the UKB under any condition. In the original GWAS, Yengo and colleagues reported height prediction accuracy using traditional PGS approaches of R^2^ = ∼0.40–0.44 in individuals of European ancestry and R^2^ = ∼0.13–0.20 in a Latino/Hispanic group.^8^ We applied these existing polygenic risk scores for height to the HCHS/SOL, an independent Latino/Hispanic cohort with no overlap with the cohort used by Yengo et al., and observed similar results (R^2^ = 0.218). We did not apply this existing PGS to our UKB EUR cohort because UKB data were included in the Yengo et al. training dataset, which would lead to overfitting, while the HCHS/SOL was not included in the training set. Moreover, other groups have used less traditional PGS approaches, such as non-linear machine learning, and report that a roughly 22% height variance is explained in their diverse Latino group.^34^ Using Lasso and ridge regression alone, we achieved prediction accuracy of about 0.19–0.25 in the HCHS/SOL and 0.25–0.37 in UKB EUR (depending on *p* value thresholds). Notably, our results in the UKB cohort did not reach the 44% accuracy observed in the most recent Yengo et al. GWAS, most likely due to the lower sample size in our UKB training cohort (*n* = 367,059) compared to that of the Yengo et al. GWAS^8^ (*n* = 3.5 million). While Lasso regression improved prediction accuracy in the HCHS/SOL compared to other studies,^6^^,^^8^^,^^34^ there remains significant potential to refine PGSs to improve accuracy in diverse populations, as current methods still perform better in European populations than in non-European groups.

Previous research within the HCHS/SOL assessed the generalizability of PGSs from European populations to Hispanics/Latinos.^6^ The study evaluated PGS performance using various strategies for SNP selection and weighting, including effect sizes estimated from large European GWASs and an admixed training population (HCHS/SOL), as well as from a fixed-effects meta-analysis combining both European and HCHS/SOL GWAS results. For height, PGSs constructed using meta-analysis-derived effect sizes explained the highest proportion of variance (12%) in Hispanics/Latinos from the Women’s Health Initiative (WHI; *n* = 3,582).^6^ There are a couple of differences between our study and this one. First, we utilized the most recent and largest height GWAS by Yengo et al.^8^ to select SNPs, which included a sample size of 3.5 million participants for SNP selection, whereas this study relied on the Wood et al. (2014) GWAS,^37^ which had a sample size of 253,288. The larger sample size in our study may enhance the statistical power and precision of selecting SNPs, potentially leading to improved PGS performance in the Hispanic/Latino population. We also estimated effect sizes in the UK using a cohort that included both European and non-European participants, and we validated our results in the HCHS/SOL, not the WHI. Using Lasso and ridge regression alone, we achieved a prediction accuracy (R^2^) of approximately 0.19–0.25 in the HCHS/SOL and an R^2^ of 0.218 when applying the most recent published Yengo GWAS PRS weights to HCHS/SOL. This is comparable to Yengo et al.’s findings in Hispanic/Latino populations, which reported an R^2^ of 0.13–0.20. Despite these differences, Grinde et al. found that selecting variants based on European GWASs generally performs well; however, estimating SNP weights using non-European GWASs improved prediction accuracy. Notably, our analyses used only European GWAS summary statistics, and we observed improved generalizability when ancestry was explicitly modeled in the target cohort.

The main contribution of our study lies in modeling PCs with PGSs within our Latino cohort, which improved prediction accuracy by approximately 5%–7% compared to PGSs alone. Deriving PCs is relatively straightforward and computationally efficient. In the HCHS/SOL, models trained in the European-only cohort performed slightly better than those trained in a more heterogeneous cohort when ancestry was not modeled (PGS only), most likely reflecting residual stratification that was not removed prior to PGS construction. Once genetic ancestry was incorporated into the prediction model, performance became comparable across training cohorts. These findings indicate that increasing ancestry diversity in the training data alone may be insufficient and that explicit ancestry modeling improves generalizability to admixed populations. Given that PGSs trained in European-only cohorts performed better when applied to individuals with predominantly European ancestry, this also highlights the need for recruitment strategies that ensure training cohorts are representative of the ancestry composition of the target population—rather than simply more diverse—to improve generalizability.

Further examination of ancestry-predominant subgroups within the HCHS/SOL revealed differences in how prediction improved with inclusion of PCs in the model (Figure S5). When using PGSs alone, prediction accuracy remained higher in the European-predominant subgroup; the Amerindian-predominant subgroup exceeded the European-predominant subgroup only after the inclusion of PCs. The larger improvement in R^2^ observed in the Amerindian-predominant subgroup after inclusion of PCs may reflect greater phenotypic variance associated with ancestry-related structure in this admixed population. Inclusion of PCs therefore increases total explained variance more strongly in this subgroup. In contrast, the European-predominant subgroup likely has less ancestry-related variance in height that can be captured by PCs, resulting in a smaller incremental gain in R^2^ when ancestry structure is explicitly modeled.

This study has several limitations. First, the small sample size in the HCHS/SOL cohort, especially within different ancestral subgroups, limits the statistical power of our subgroup analyses within different predominant ancestry groups. Second, our analysis focused on height, since it is a highly heritable and well-studied trait. However, it is crucial to apply our approach to a broader range of heritable traits and diseases to assess its generalizability to health-related outcomes. Additionally, the generalizability of our findings may be limited by the specific methodologies used for PGSs and the choice of prediction models, such as Lasso and ridge regression. Lasso regression, while effective, is sensitive to missing values and may be less robust when replicating findings in other cohorts with varying degrees of missing data. To mitigate the impact of these limitations, we included SNPs that were shared across our GWAS, training, and validation cohorts. We also included the most recent height PGS from Yengo et al. as a benchmark and contextualized our results relative to prior studies.

Another limitation is that our approach accounted for ancestry using PCs, which captures broad patterns of population structure but may not fully represent local ancestry effects. Prior work suggests that PCs primarily reflect continental-scale variation^38^ and may average out fine-scale ancestry signals that are relevant to genetic prediction, particularly in admixed populations such as the HCHS/SOL.^39^ Consistent with this, we observed that predictive gains were largely concentrated in the first few PCs, with minimal improvement beyond PC3. In addition, PCs may capture non-genetic or environmental factors that are correlated with ancestry, which could contribute to the variance explained by prediction models and complicate the interpretation of ancestry-related effects. Incorporating local ancestry inference in future work could help identify ancestry-specific genetic effects that are not captured by global PCs and may further enhance prediction accuracy in admixed populations. Addressing these limitations will be crucial to refining polygenic prediction models and improving their applicability across diverse traits and populations.

In summary, explicitly modeling ancestry information with PCs in PGS models improves the generalizability of PGSs to admixed populations, with our study demonstrating that adding PCs increased prediction accuracy by approximately 5%. Despite this advancement, prediction accuracy for height in admixed populations, such as the HCHS/SOL, remains below that achieved in a European ancestry cohort. Therefore, increasing recruitment efforts in diverse populations remains essential to enhance the generalizability and effectiveness of polygenic prediction models.

## Data and code availability

The UKB data used in this study are available through the UKB data portal (https://www.ukbiobank.ac.uk/use-our-data/apply-for-access/). Data from the HCHS/SOL can be accessed through the study’s data coordinating center following their established procedures (https://sites9.cscc.unc.edu/hchs/home). GWAS summary statistics used for SNP selection were obtained from the GIANT Consortium (https://giant-consortium.web.broadinstitute.org/index.php/GIANT_consortium_data_files).

All code used for data processing, analysis, and figure generation is available on GitHub at https://github.com/xinwangsuperbrain/Genetic_ancestry_prediction.git.

## Acknowledgments

This research has been conducted using data from the UKB, a major biomedical database, under application number 27412. The authors also thank the staff and participants of the Hispanic Community Health Study/Study of Latinos (HCHS/SOL) for their important contributions to the HCHS/SOL studies. The HCHS/SOL is a collaborative study supported by contracts from the 10.13039/100000050National Heart, Lung, and Blood Institute (NHLBI) to the University of North Carolina (HHSN268201300001I/N01-HC-65233), 10.13039/100006686University of Miami (HHSN268201300004I/N01-HC-65234), 10.13039/100007319Albert Einstein College of Medicine (HHSN268201300002I/N01-HC-65235), 10.13039/100008522University of Illinois at Chicago (HHSN268201300003I/N01-HC-65236, Northwestern University), and 10.13039/100007099San Diego State University (HHSN268201300005I/N01-HC-65237). The following institutes/centers/offices have contributed to the HCHS/SOL through a transfer of funds to the NHLBI: the National Institute on Minority Health and Health Disparities, the 10.13039/100000055National Institute on Deafness and Other Communication Disorders, the 10.13039/100000072National Institute of Dental and Craniofacial Research, the 10.13039/100000062National Institute of Diabetes and Digestive and Kidney Diseases, the 10.13039/100000065National Institute of Neurological Disorders and Stroke, and the NIH Institution-Office of Dietary Supplements.

## Declaration of interests

A.M.D. is the founding director and holds equity in CorTechs Labs, Inc. (DBA Cortechs.ai) and serves on its board of directors and the scientific advisory board. He is an unpaid consultant for Oslo University Hospital. The terms of these arrangements have been reviewed and approved by the University of California, San Diego, in accordance with its conflict-of-interest policies.

## Supplemental information

Document S1. Figures S1–S8 and Tables S1 and S2

Document S2. Article plus supplemental information
