# Supplementary material for: Explicitly modeling genetic ancestry to improve polygenic prediction accuracy for height in a large, admixed cohort of US Latinos: Findings from HCHS/SOL
Source: HGG Adv. 2026 Mar 27;7(3):100597. doi: 10.1016/j.xhgg.2026.100597 (PMC13157043; doi:10.1016/j.xhgg.2026.100597)
Supplement: Document S1. Figures S1–S8 and Tables S1 and S2 [file mmc1.pdf]

HGGA, Volume 7

Supplemental information

Explicitly modeling genetic ancestry to improve polygenic prediction accuracy for height in a large, admixed cohort of US Latinos: Findings from HCHS/SOL

Xin Wang, Tamar Sofer, Oleksandr Frei, Robert Kaplan, Krista M. Perreira, Nora Franceschini, Humberto Parada Jr., Laura Zhou, Ole A. Andreassen, Hector Gonzalez, Anders M. Dale, and Iris J. Broce

Supplementary

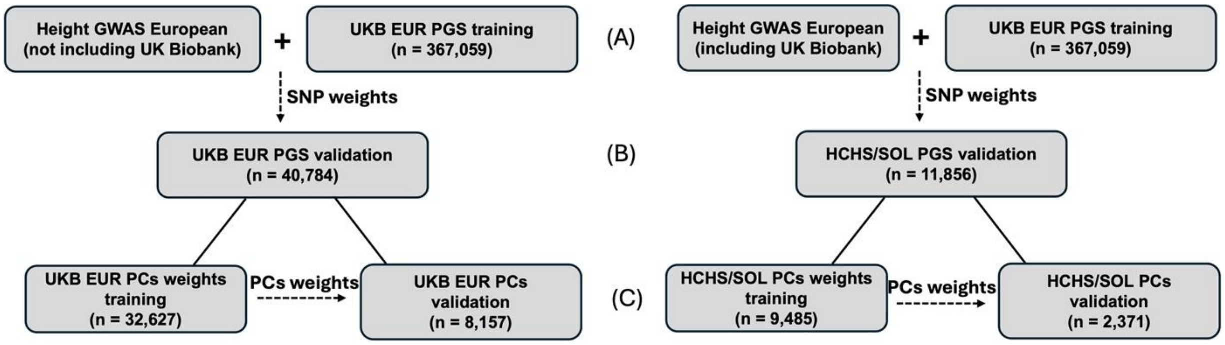

Supplementary Figure 1. Analytic diagram in the main analyses and sample size used to train and test height prediction in UKB EUR and HCHS/SOL

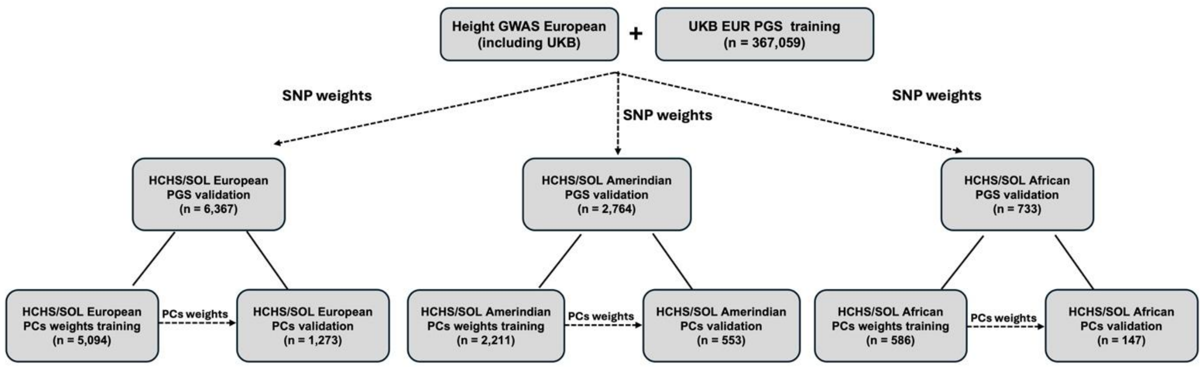

Supplementary Figure 2. Analytic diagram and sample size used to train and test height prediction using UKB EUR-trained PGS in different ancestry subsets of HCHS/SOL

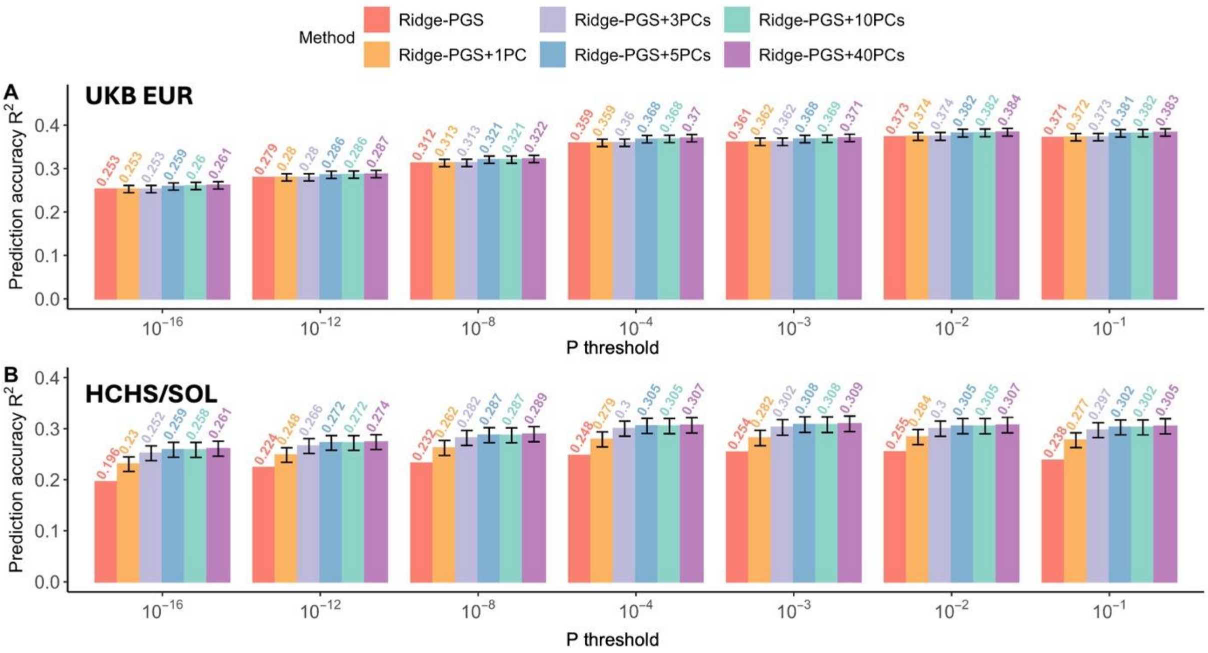

Supplementary Figure 3. Predictive performance comparison on height between PGS only and combination of PGS and PCs using Ridge regression among UKB European and HCHS/SOL. Error bars indicate standard deviation.

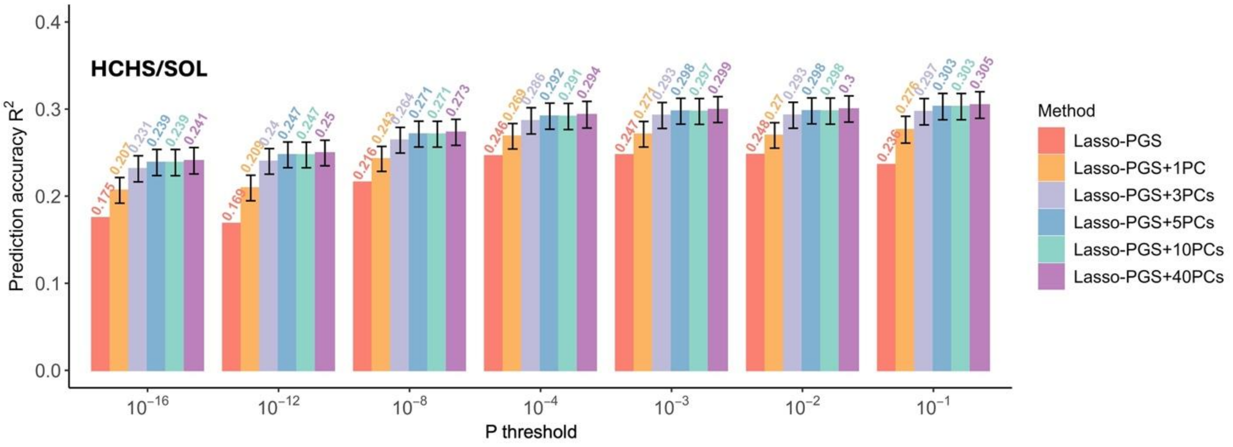

Supplementary Figure 4. Predictive performance comparison on height between PGS only and combination of PGS and PCs using Lasso regression among HCHS/SOL. Prediction analyses in HCHS/SOL were conducted using GWAS summary statistics that exclude UK Biobank. Error bars indicate standard deviation.

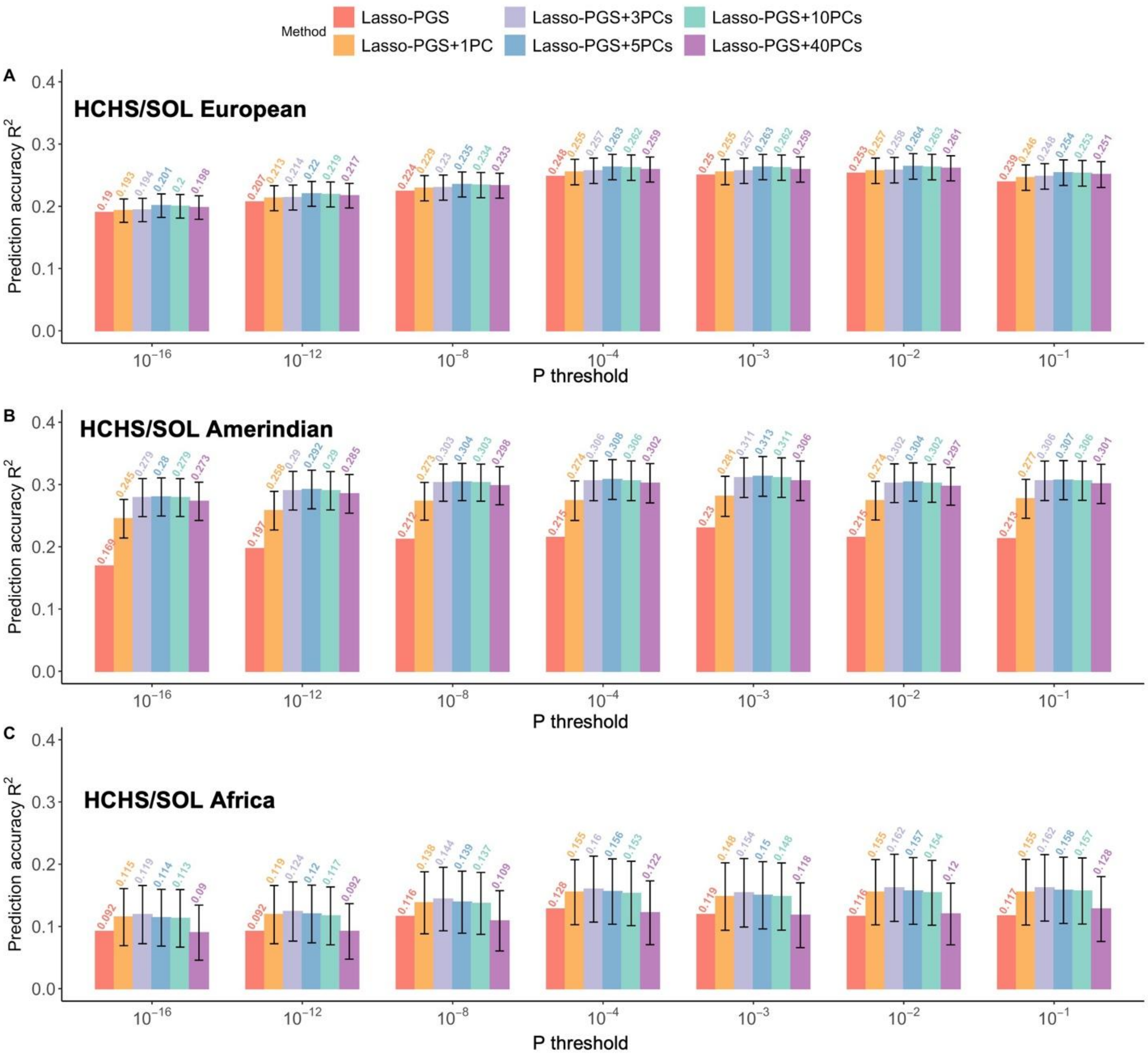

Supplementary Figure 5. Predictive performance comparison on height between UKB European-trained PGS only and combination of PGS and PCs using Lasso regression in different ancestry subsets of HCHS/SOL European (A), American (B) and African (C) in HCHS/SOL. Error bars indicate standard deviation.

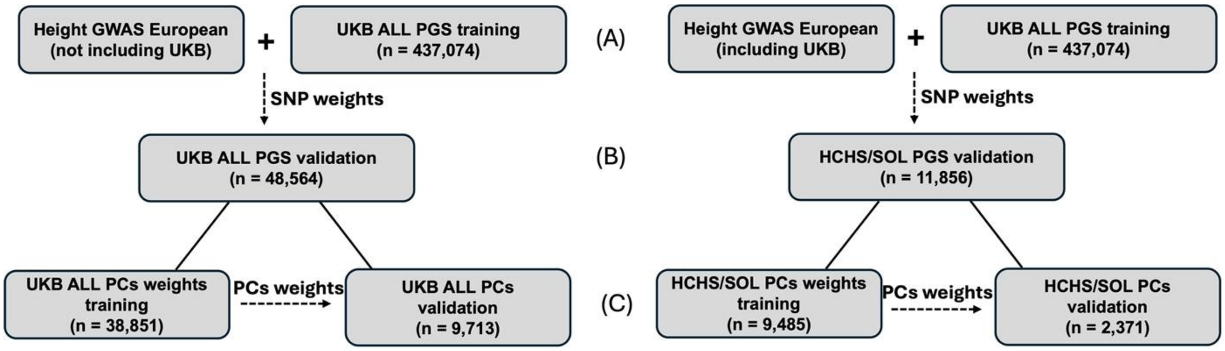

Supplementary Figure 6. Analytic diagram in the main analyses and sample size used to train and test height prediction in UKB ALL and HCHS/SOL. Error bars indicate standard deviation.

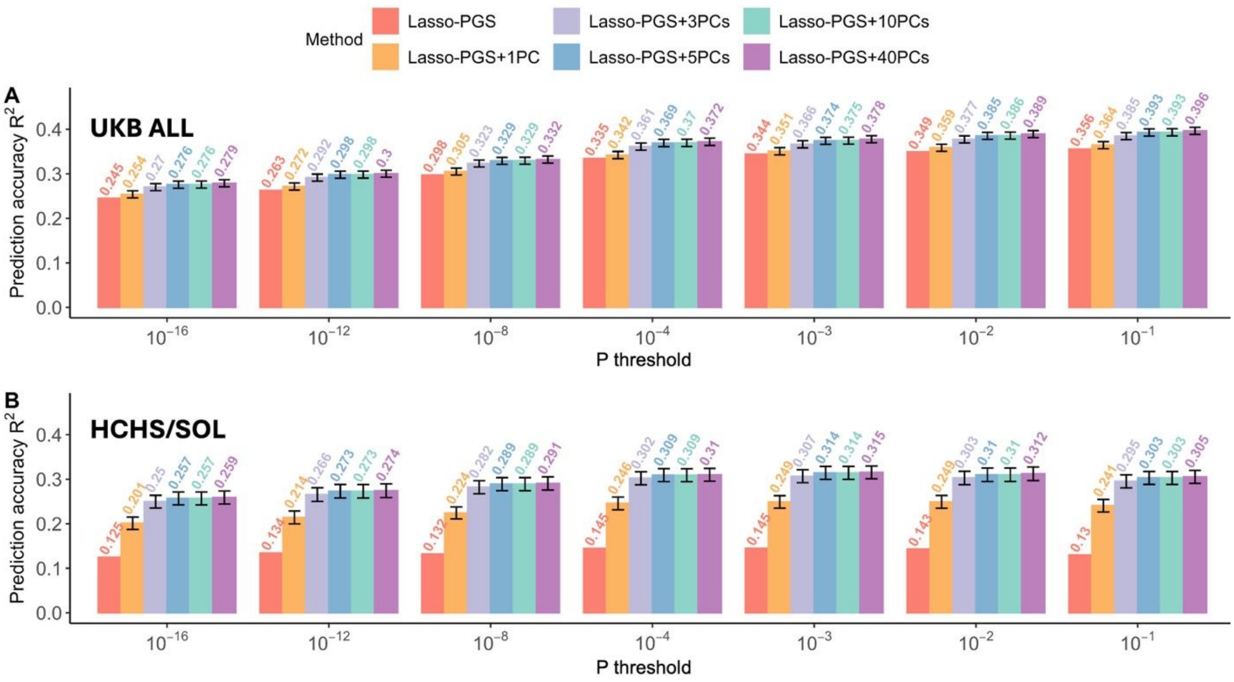

Supplementary Figure 7. Predictive performance comparison on height between UKB ALL-trained PGS only and combination of PGS and PCs using Lasso regression among UKB ALL and HCHS/SOL. Error bars indicate standard deviation.

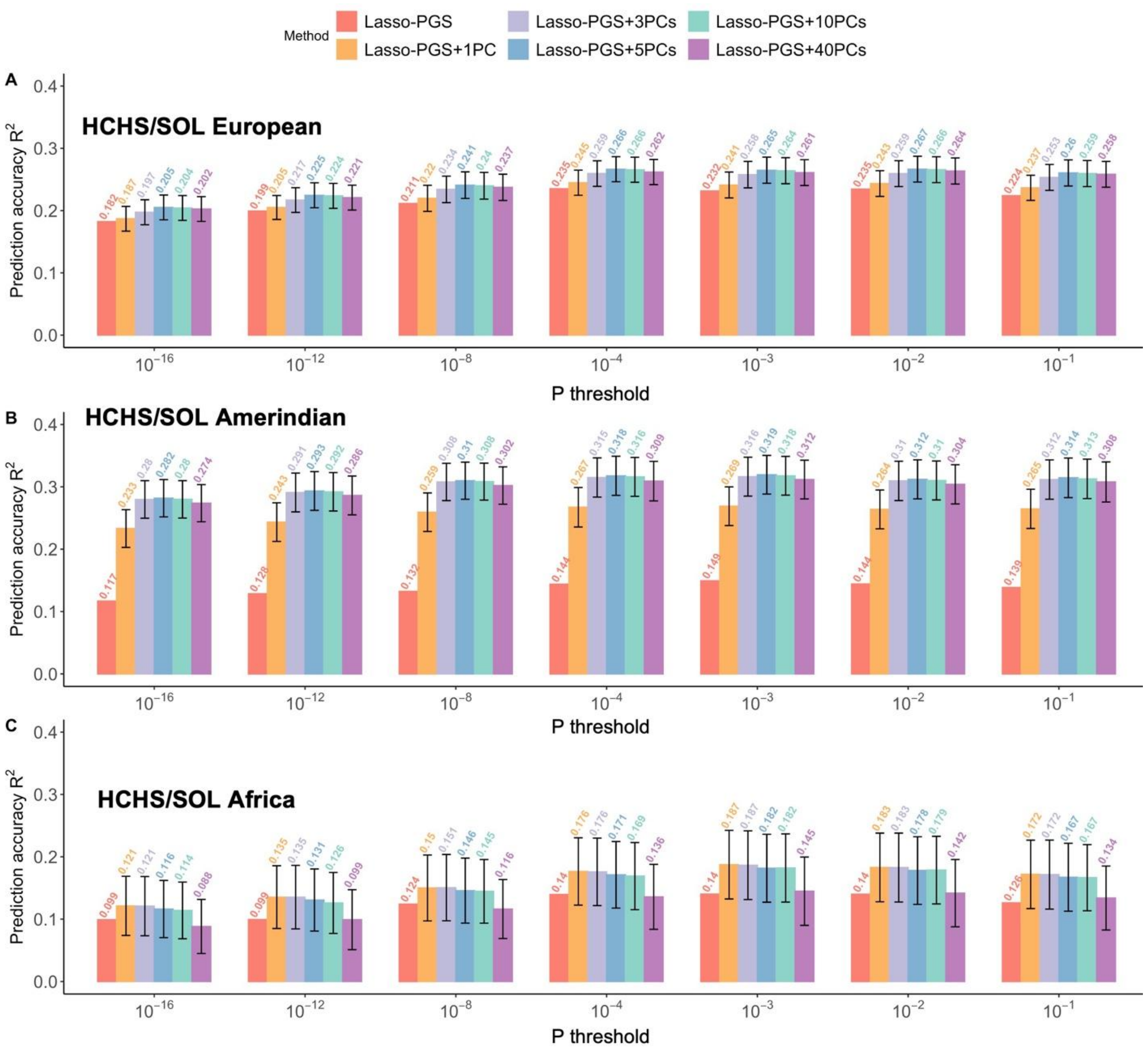

Supplementary Figure 8. Predictive performance comparison on height between UKB ALL-trained PGS only and combination of PGS and PCs using Lasso regression in different ancestry subsets of HCHS/SOL European (A), American (B) and African (C) in HCHS/SOL. Error bars indicate standard deviation.

GWAS not including UKB		GWAS including UKB	
**P threshold**	**N SNPs**	**P threshold**	**N SNPs**
$< 10^{-16}$	15,706	$< 10^{-16}$	29,625
$< 10^{-12}$	22,776	$< 10^{-12}$	40,551
$< 10^{-8}$	37,111	$< 10^{-8}$	61,300
$< 10^{-4}$	79,936	$< 10^{-4}$	117,286
$< 10^{-3}$	107,373	$< 10^{-3}$	149,883
$< 10^{-2}$	158,724	$< 10^{-2}$	206,174
$< 10^{-1}$	277,334	$< 10^{-1}$	323,550

Supplementary Table 1. Number of SNPs in the prediction with different P thresholds. No pruning was performed.

GWAS with UKB trained in UKB European-only						
P **< 10^{-1}**	***P*** **< 10^{-2}**	***P*** **< 10^{-3}**	***P*** **< 10^{-4}**	***P*** **< 10^{-8}**	***P*** **< 10^{-12}**	***P*** **< 10^{-16}**
55.4138	55.1617	55.8408	53.7971	55.6422	54.8876	55.1248
19.9147	19.8241	20.0682	19.3337	19.9968	19.7256	19.8108
7.157	7.1244	7.2121	6.9482	7.1865	7.089	7.1196
2.5721	2.5604	2.5919	2.497	2.5827	2.5477	2.5587
0.9244	0.9202	0.9315	0.8974	0.9282	0.9156	0.9195
0.3322	0.3307	0.3348	0.3225	0.3336	0.329	0.3305
0.1194	0.1188	0.1203	0.1159	0.1199	0.1183	0.1188
0.0429	0.0427	0.0432	0.0417	0.0431	0.0425	0.0427
0.0154	0.0153	0.0155	0.015	0.0155	0.0153	0.0153
0.0055	0.0055	0.0056	0.0054	0.0056	0.0055	0.0055
**GWAS with UKB trained in UKB ALL**						
P **< 10^{-1}**	***P*** **< 10^{-2}**	***P*** **< 10^{-3}**	***P*** **< 10^{-4}**	***P*** **< 10^{-8}**	***P*** **< 10^{-12}**	***P*** **< 10^{-16}**
51.8317	53.0878	51.7632	52.9919	52.7432	52.8528	51.9848
18.6274	19.0788	18.6027	19.0443	18.9549	18.9943	18.6824
6.6943	6.8566	6.6855	6.8442	6.812	6.8262	6.7141
2.4058	2.4641	2.4026	2.4597	2.4481	2.4532	2.4129
0.8646	0.8856	0.8635	0.884	0.8798	0.8816	0.8672
0.3107	0.3183	0.3103	0.3177	0.3162	0.3168	0.3116
0.1117	0.1144	0.1115	0.1142	0.1136	0.1139	0.112
0.0401	0.0411	0.0401	0.041	0.0408	0.0409	0.0402
0.0144	0.0148	0.0144	0.0147	0.0147	0.0147	0.0145
0.0052	0.0053	0.0052	0.0053	0.0053	0.0053	0.0052

Supplementary Table 2. Lambda (λ) values during Lasso regression for height prediction across various GWAS p-value thresholds. Results are shown separately for models trained using GWAS summary statistics from UK Biobank European-only and UK Biobank all ancestry samples.
